# Construction of a Multi-Label Classifier for Extracting Multiple Incident Factors From Medication Incident Reports in Residential Care Facilities: Natural Language Processing Approach

**DOI:** 10.2196/58141

**Published:** 2024-07-23

**Authors:** Hayato Kizaki, Hiroki Satoh, Sayaka Ebara, Satoshi Watabe, Yasufumi Sawada, Shungo Imai, Satoko Hori

**Affiliations:** 1 Division of Drug Informatics Keio University Faculty of Pharmacy Tokyo Japan; 2 Graduate School of Pharmaceutical Sciences The University of Tokyo Tokyo Japan; 3 Interfaculty Initiative in Information Studies The University of Tokyo Tokyo Japan

**Keywords:** residential facilities, incidents, non-medical staff, natural language processing, risk management

## Abstract

**Background:**

Medication safety in residential care facilities is a critical concern, particularly when nonmedical staff provide medication assistance. The complex nature of medication-related incidents in these settings, coupled with the psychological impact on health care providers, underscores the need for effective incident analysis and preventive strategies. A thorough understanding of the root causes, typically through incident-report analysis, is essential for mitigating medication-related incidents.

**Objective:**

We aimed to develop and evaluate a multilabel classifier using natural language processing to identify factors contributing to medication-related incidents using incident report descriptions from residential care facilities, with a focus on incidents involving nonmedical staff.

**Methods:**

We analyzed 2143 incident reports, comprising 7121 sentences, from residential care facilities in Japan between April 1, 2015, and March 31, 2016. The incident factors were annotated using sentences based on an established organizational factor model and previous research findings. The following 9 factors were defined: procedure adherence, medicine, resident, resident family, nonmedical staff, medical staff, team, environment, and organizational management. To assess the label criteria, 2 researchers with relevant medical knowledge annotated a subset of 50 reports; the interannotator agreement was measured using Cohen κ. The entire data set was subsequently annotated by 1 researcher. Multiple labels were assigned to each sentence. A multilabel classifier was developed using deep learning models, including 2 Bidirectional Encoder Representations From Transformers (BERT)–type models (Tohoku-BERT and a University of Tokyo Hospital BERT pretrained with Japanese clinical text: UTH-BERT) and an Efficiently Learning Encoder That Classifies Token Replacements Accurately (ELECTRA), pretrained on Japanese text. Both sentence- and report-level training were performed; the performance was evaluated by the *F*_1_-score and exact match accuracy through 5-fold cross-validation.

**Results:**

Among all 7121 sentences, 1167, 694, 2455, 23, 1905, 46, 195, 1104, and 195 included “procedure adherence,” “medicine,” “resident,” “resident family,” “nonmedical staff,” “medical staff,” “team,” “environment,” and “organizational management,” respectively. Owing to limited labels, “resident family” and “medical staff” were omitted from the model development process. The interannotator agreement values were higher than 0.6 for each label. A total of 10, 278, and 1855 reports contained no, 1, and multiple labels, respectively. The models trained using the report data outperformed those trained using sentences, with macro *F*_1_-scores of 0.744, 0.675, and 0.735 for Tohoku-BERT, UTH-BERT, and ELECTRA, respectively. The report-trained models also demonstrated better exact match accuracy, with 0.411, 0.389, and 0.399 for Tohoku-BERT, UTH-BERT, and ELECTRA, respectively. Notably, the accuracy was consistent even when the analysis was confined to reports containing multiple labels.

**Conclusions:**

The multilabel classifier developed in our study demonstrated potential for identifying various factors associated with medication-related incidents using incident reports from residential care facilities. Thus, this classifier can facilitate prompt analysis of incident factors, thereby contributing to risk management and the development of preventive strategies.

## Introduction

The prevention of medication-related incidents and the development of preventive measures are crucial for ensuring medication safety. Heinrich law suggests that for every serious accident, 29 minor accidents and 300 incidents exist [1]. Analysis of these incidents and formulation of countermeasures can help prevent serious medical accidents and enhance patient safety. Moreover, these incidents result in a significant psychological impact on the health care providers [2-5], known as “second victim syndrome” [6-8]. Thus, incident prevention measures are considered vital.

The core of incident prevention is focused on the details of the incident; thus, the creation of incident reports plays a key role. Hospitals have traditionally been the primary sources of such data, resulting in extensive research [9-19] and the development of sophisticated incident prevention strategies. However, residential care facility settings, in which residents live for extended periods, present unique challenges. Unlike hospitals, these facilities serve as communal living spaces for older people and often rely on nonmedical staff (not doctors or nurses) to perform health care–related tasks, including medication assistance. This practice raises significant concerns regarding the potential for medication incidents, underlining the importance of extending incident prevention strategies beyond hospital settings. Moreover, previous studies have highlighted a range of medication incidents in Japanese residential care facilities, including dropped drugs and misdelivery or misuse of medicines [20].

Natural language processing (NLP) technology demonstrates considerable potential for enhancing the analysis of incident reports. NLP is an analytical technique involving deep learning processing of human language for extracting meaningful information. Recently, this technology has been applied to classify various types of text data, including blogs [21,22] and electronic medical records [23], and has been extended to incident report classification in health care settings [24,25]. Specifically, classifiers using NLP were constructed to determine the classification and severity of incidents, with hospital incident reports serving as the training data [24,26,27]. Although the incident reports obtained and their corresponding text data, primarily comprising open-ended descriptions, from residential care facilities are considered suitable for NLP analysis, limited efforts have been made toward using NLP for extracting information from incident reports at these facilities.

Our previous research, which focused on identifying the causes of medication incidents in residential care facilities, demonstrated the complex and multifactorial nature of various elements contributing to these incidents [28]. Therefore, this study aimed to create a multilabel classifier that can extract various factors related to medication-related incidents based on incident reports in residential care facilities.

## Methods

### Data Set

This study included incidents that occurred in residential care facilities in Japan from April 1, 2015, to March 31, 2016, in 106 long-term residential care facilities operated by a single company. The residential care facilities included in our study are privately run, where residents usually pay monthly fees for housing and various care or support services, including meal provision, assistance with activities of daily living, and recreational opportunities. Notably, the majority of the residents avail medication assistance, which is a crucial component of these services.

We exclusively focused on incidents involving care staff who were not medical professionals. An incident report was completed after each incident, documenting the type of incident (forgetting to take medicines, misdelivery or misuse of medicines, loss of medicines, discovery of dropped drugs, and spitting up or falling while taking medicines), conditions at the time, and factors contributing to the incident. All the reports were written in Japanese. The care staff at the participating facilities were encouraged to record even minor incidents in their reports.

The data set comprised 2143 reports. The free-text descriptions of the factors contributing to the incident in each report were segmented into 7121 sentences for further analysis.

### Annotation and Data Analysis

Incident factor labels were established based on the organizational factor model by Reason [29] and findings from our previous study [28], which explored the factors of medication assistance-related incidents in residential care facilities. In our previous study, we interviewed individuals involved in incidents, such as misdelivery or misuse of medications. Our findings indicated that “not following procedures” often resulted in these incidents, and identified 4 key contributing factors, namely individual residents, individual staff, team, and work environment. Considering the diverse nature of incidents in residential care facilities that extend beyond medication omissions or dropped drugs, a broader range of factor labels is warranted. We developed the following 9 causal labels: procedure adherence, medicine, resident, resident family, nonmedical staff, medical staff, team, environment, and organizational management. To establish the labels, we also consulted James Reason [29] organizational accident model. Reason model posits that incidents, which are often precipitated by unsafe acts attributed to multiple factors, are fundamentally rooted in the culture of the organization. This model provides a framework for understanding the complex interplay of the factors resulting in incidents in our study.

The criteria for annotating the reports were based on our previous study [28] and an analysis of actual medication incident conditions in Japan [20]. To evaluate the reliability of these criteria, we selected a random sample of 50 reports comprising 183 sentences from the data set. These reports were annotated by 2 researchers with relevant medical knowledge (HK and SE). The interannotator agreement (IAA) was assessed using Cohen κ, a statistical measure of agreement. Cohen Κ values are interpreted as follows: values close to 1 indicate perfect agreement, <0.00, “poor”; 0.00-0.20, “slight”; 0.21-0.40, “fair”; 0.41-0.60, “moderate”; 0.61-0.80, “substantial”; and 0.81-1.00, “almost perfect” [30]. Following this initial assessment, one of the researchers (HK) annotated all the sentences.

The distribution of reports according to the number of labels assigned was analyzed. The average number of labels per incident type was calculated and compared. Incident types were categorized as “forgetting to take medicines,” “misdelivery or misuse of medicines,” “loss of medicines,” “discovery of dropped drugs,” and “spitting up or falling while taking medicines,” as defined in our previous study [20]. For factor analysis, we used the Student *t* test (2-tailed), applying the Bonferroni correction to account for multiple comparisons. We established the significance criterion at *P*<.05. Furthermore, the percentage of reports that contained each label for every type of incident was calculated, thus providing a detailed view of the label distribution across different incident categories.

### Deep Learning Models

**Figure 1:**
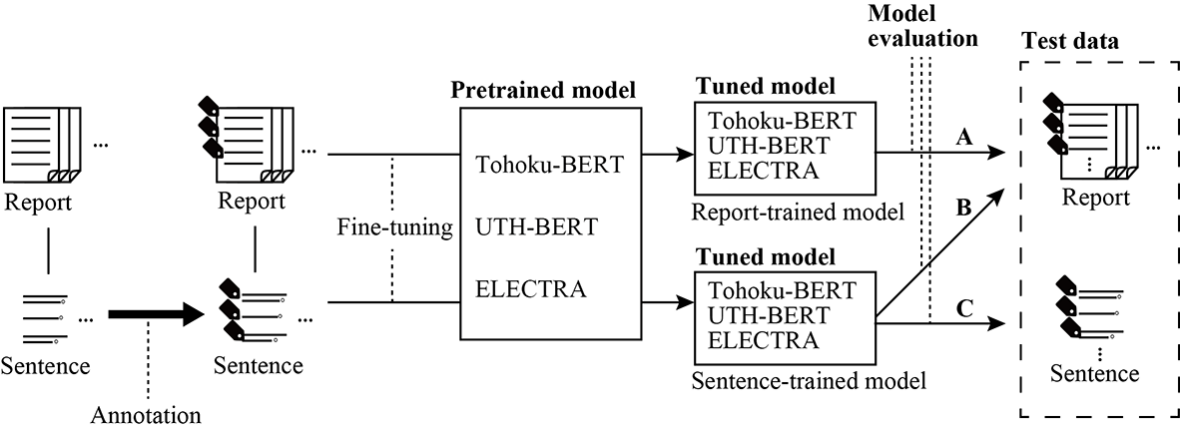
Overview of model development and evaluation. (A) Report-trained model evaluated using reports. (B) Sentence-trained model evaluated using reports. (C) Sentence-trained model evaluated using sentences. BERT: Bidirectional Encoder Representations From Transformers; ELECTRA: Encoder That Classifies Token Replacements Accurately; UTH: University of Tokyo Hospital.

Figure 1 shows an overview of the model development. In this study, a multilabel classifier was built from an annotated multilabel data set to manage multiple descriptions of incident factors. Due to the insufficient number of labels, which made the accurate evaluation of the model’s performance challenging, the limited labels associated with “resident family” and “medical staff” were excluded from the model development process. Consequently, the refined model development approach enabled the classifier to simultaneously identify 7 distinct labels. The development of this classifier involves fine-tuning existing pretrained models. These models included 2 Bidirectional Encoder Representations From Transformers (BERT) models, each using different pretraining data sources, and an Efficiently Learning Encoder That Classifies Token Replacements Accurately (ELECTRA) model. ELECTRA, while maintaining a foundational structure similar to that of BERT, achieves enhanced performance in NLP tasks through improved pretraining methods. The input of these models was limited to 512 tokens due to the capability of the pretrained model.

Specifically, one of the BERT models we used was developed by the Natural Language Processing Research Group at Tohoku University (Tohoku-BERT), which was pretrained on the Japanese Wikipedia data as of September 1, 2019 [31] (BERT-based model; 12 layers, 768 dimensions of hidden states, and 12 attention heads, tokenizer: MeCab [32]). The other, University of Tokyo Hospital (UTH)-BERT, was developed by the Department of Artificial Intelligence and Digital Twin in Healthcare at the University of Tokyo and pretrained using extensive Japanese clinical text [33] (BERT-based model: 12 layers, 768 dimensions of hidden states, and 12 attention heads, tokenizer: MeCab [32]). The ELECTRA model was developed by the Izumi Laboratory at the University of Tokyo and pretrained with Japanese Wikipedia data as of June 1, 2021 [34] (ELECTRA-based model; 12 layers, 768 dimensions of hidden states, and 12 attention heads, tokenizer: MeCab [32]). No additional preprocessing was conducted beyond what is described.

Our study used 2 distinct models: one based on reports and the other on sentences. In the report-trained model, each report served as a single training unit, whereas in the sentence-trained model, each sentence served as a training unit.

The hyperparameters that can be adjusted before training are defined in Table S1 in Multimedia Appendix 1.

### Task and Metrics

Performance was evaluated in terms of precision, recall, *F*_1_-score, and exact match accuracy. The exact match accuracy specifically measures the percentage of predictions that are correct across all labels. The data set was divided into training and test data at a ratio of 4:1, and the model was evaluated using the average of the 5-fold cross-validation results.

The report-trained model was evaluated using the reports as test data (Figure 1A). The sentence-trained model was evaluated using reports (Figure 1B) and sentences (Figure 1C) as test data.

### Generalizability Analysis

We extracted 136 incident reports involving nonmedical staff and 31 reports involving care staff from hospital incident data collected by the Japan Council for Quality Health Care between January 2010 and June 2023 to examine the generalizability of the constructed model. We assessed the ability for extrapolation of the report-trained model derived from the extractor pretrained on Tohoku-BERT using the *F*_1_-score.

### Ethical Considerations

All the procedures were performed per the principles of the Declaration of Helsinki. In this study, all data were analyzed anonymously, and informed consent was waived owing to the retrospective observational design of this study. Residents and staff in residential facilities were informed of this study through postings at each facility and were allowed to refuse permission concerning the use of their data. This study was approved by the Research Ethics Review Committee of the Faculty of Pharmaceutical Sciences, University of Tokyo (approved on August 3, 2023) and the Research Ethics Review Committee of the Faculty of Pharmacy, Keio University (approved on July 14, 2023; 230714-1).

## Results

### Data Set Analysis

The average report length was 62.6 (SD 34.3; median 56, IQR 38-81) tokens, and the average sentence length was 18.2 (SD 9.8; median 16, IQR 11-23) tokens. None of the sentences or reports exceeded 512 tokens. The incidents were categorized as follows: forgetting to take medicines (648 incidents), misdelivery or misuse of medicines (293 incidents), loss of medication (18 incidents), discovery of dropped drugs (1024 incidents), and spitting up or falling while taking medicines (160 incidents).

### Annotation and Features of the Incident Factors

An example of this label is presented in Textbox 1. The IAA for each label was calculated, and all the labels achieved an IAA score exceeding 0.6 (Table 1), thereby validating the effectiveness of the developed annotation guidelines. Notably, the κ coefficients for the factors related to “resident family” and “team” were exceptionally high, exceeding 0.9 in all instances. Using these guidelines, the remaining reports were sequentially annotated. The “resident” related factor label was most frequently assigned as shown in Table 1. Conversely, the “resident family” and “medical staff” factors were rarely assigned.

Table 2 presents the distribution of the number of labels assigned per sentence and per report. The most frequent occurrences were 1 label per sentence and 2 labels per report, accounting for 77.5% (5518) sentences and 34.2% (733) reports of the total occurrences, respectively. Table 3 categorizes the number of labels per report according to the incident type. Reports involving forgetting to take medicines and misdelivery or misuse of medicines tended to have a higher number of labels than other incidents (*P*<.001).

Table 4 shows the percentage of incident reports, with each label categorized by the incident type. Reports describing incidents, such as “spitting up or falling while taking medicines” and “discovery of dropped drugs,” often included “resident” factors and less frequently mentioned “team” factors. In contrast, reports of “forgetting to take medicines” and “misdelivery or misuse of medicines” commonly included “environmental” factors.

**Example of the label.**

**Procedure adherence**

- Care staff did not follow the instructions for double-checking medication assistance.
- Care staff failed to confirm that medications were swallowed until the end.

**Medicine**

- Due to the concurrent use of herbal medicine with pills, there was a higher risk of dropping them.
- The number of medications to be taken after breakfast was large.

**Resident**

- The resident was unable to manage their medications.
- Their life rhythm was irregular, with mealtimes being inconsistent.

**Resident family**

- Family members were assisting with meals, which prevented intervention in medication administration.
- Medication management was being handled by the family.

**Nonmedical staff**

- Preparation for breakfast was not sufficient, leading to delays in service time and causing staff to rush.
- There was a low awareness that numbness made it difficult for residents to hold medication packets.

**Medical staff**

- Inexperienced nurses relied on each other, resulting in a lack of necessary checks.
- The nursing notes failed to include the required documentation.

**Team**

- There was a lack of coordination between meal assistance and medication assistance staff.
- Important information from doctor visits was not properly communicated.

**Environment**

- The resident was taking medicines during the busiest time for medication assistance.
- The proximity of tables in the restaurant made it impossible to check medication intake.

**Organizational management**

- Measures against the previous incidents had not been implemented.
- The procedure manual had not been updated.

**Table 1:** The IAA^a^ values and number of sentences for the 9 labels (N=7121).

Label	IAA	Sentences, n
Procedure adherence	0.634	1167
Medicine	0.741	694
Resident	0.898	2455
Resident family	0.954	23
Nonmedical staff	0.638	1905
Medical staff	0.869	46
Team	0.930	195
Environment	0.755	1104
Organizational management	0.692	195

^a^IAA: interannotator agreement.

**Table 2:** The number of labels per incident report (N=2143) and per sentence (N=7121).

Number of labels	Reports, n (%)	Sentences, n (%)
0	10 (0.5)	508 (7.1)
1	278 (13.0)	5518 (77.5)
2	733 (34.2)	1021 (14.3)
3	689 (32.2)	70 (1.0)
4	348 (16.2)	3 (0.04)
5	78 (3.6)	0 (0)
6	7 (0.3)	0 (0)
≥7	0 (0)	0 (0)

**Table 3:** The average number of labels per incident contents.

	Labels, mean (SD)
Forgetting to take medicines (n=648)	2.92 (1.10)
Misdelivery or misuse of medicines (n=293)	2.85 (1.12)
Loss of medicines (n=18)	2.61 (1.38)
Discovery of dropped drugs (n=1024)	2.45 (0.94)
Spitting up or falling while taking medicines (n=160)	2.19 (0.93)

**Table 4:** The percentages of reports that contain each label for every type of incident.

	Procedure adherence	Medicine	Resident	Resident family	Nonmedical staff	Medical staff	Team	Environment	Organizational management
Forgetting to take medicines (n=648)	41.0	33.0	59.0	2.3	72.8	2.8	17.0	53.4	11.0
Misdelivery or misuse of medicines (n=293)	39.6	28.7	59.7	0.7	73.0	4.8	14.0	53.6	11.3
Loss of medicines (n=18)	27.8	33.3	61.1	0	61.1	0	11.1	38.9	27.8
Discovery of dropped drugs (n=1024)	57.0	20.3	84.3	0.3	50.2	0	0.8	25.9	6.2
Spitting up or falling while taking medicines (n=160)	12.5	22.5	81.3	0	73.1	0.6	1.9	24.4	2.5

### Model

Table S2 in Multimedia Appendix 1 shows the average label distributions for both the training and test data as part of the 5-fold cross-validation process. Since the labels for “resident family” and “medical staff” are notably fewer than those of other categories, they were excluded from the development of the multilabel classifier.

The performances of the fine-tuned Tohoku-BERT, UTH-BERT, and ELECTRA models were assessed using 3 different approaches: a report-trained model evaluated using reports (Table 5), sentence-trained model evaluated using reports (Table 6), and sentence-trained model evaluated using sentences (Table 7), as summarized in Tables 5-7. The analysis revealed that the report-trained model (Table 5) generally achieved higher *F*_1_-scores than the sentence-trained model evaluated using the report data (Table 6). The performance of the sentence-trained model was better when evaluated using sentences (Table 7) than when evaluated using reports (Table 6).

Table 8 lists the exact match accuracies of these models across the board, specifically for instances involving multiple labels. The sentence-trained model evaluated using sentences exhibited the highest exact match accuracy for the overall test data. When limited to the test data with multiple labels, the report-trained model evaluated using reports demonstrated the highest exact match accuracy.

The extrapolation of the report-trained model, fine-tuned using Tohoku-BERT, revealed that the mean *F*_1_-score (micro *F*_1_-score) was 0.72 for reports involving care staff alone and 0.65 for those involving nonmedical staff (Table S1 in Multimedia Appendix 2).

**Table 5:** Performance of the model. Report-trained model evaluated using reports.

Class	Tohoku-BERT^a^				UTH^b^-BERT				ELECTRA^c^		
	Precision	Recall	*F*_1_-score	Precision		Recall	*F*_1_-score	Precision		Recall	*F*_1_-score
Procedure adherence	0.808	0.834	0.820	0.774		0.856	0.816	0.818		0.804	0.804
Medicine	0.734	0.804	0.766	0.734		0.690	0.710	0.718		0.764	0.734
Resident	0.902	0.964	0.932	0.932		0.950	0.942	0.920		0.944	0.930
Nonmedical staff	0.802	0.852	0.820	0.778		0.842	0.806	0.822		0.852	0.832
Team	0.834	0.598	0.674	0.650		0.378	0.446	0.836		0.568	0.662
Environment	0.782	0.840	0.808	0.760		0.768	0.764	0.764		0.836	0.792
Organizational management	0.598	0.322	0.390	0.334		0.218	0.244	0.432		0.402	0.392
Macro *F*_1_-score	—^d^	—	0.744	—		—	0.675	—		—	0.735

^a^BERT: Bidirectional Encoder Representations From Transformers.

^b^UTH: University of Tokyo Hospital.

^c^ELECTRA: Encoder That Classifies Token Replacements Accurately.

^d^Not applicable.

**Table 6:** Performance of the model. Sentence-trained model evaluated using reports.

Class	Tohoku-BERT^a^				UTH^b^-BERT				ELECTRA^c^		
	Precision	Recall	*F*_1_-score	Precision		Recall	*F*_1_-score	Precision		Recall	*F*_1_-score
Procedure adherence	0.910	0.376	0.528	0.922		0.382	0.540	0.940		0.444	0.602
Medicine	0.926	0.316	0.466	0.854		0.664	0.746	0.942		0.176	0.296
Resident	0.992	0.444	0.614	0.982		0.726	0.834	0.988		0.494	0.656
Nonmedical staff	0.918	0.686	0.784	0.952		0.382	0.544	0.962		0.468	0.630
Team	1.000	0.274	0.424	0.850		0.148	0.242	0.800		0.084	0.146
Environment	0.976	0.468	0.636	0.964		0.366	0.532	0.930		0.466	0.620
Organizational management	1.000	0.064	0.116	0.600		0.018	0.034	0.884		0.068	0.126
Macro *F*_1_-score	—^d^	—	0.610	—		—	0.496	—		—	0.439

^a^BERT: Bidirectional Encoder Representations From Transformers.

^b^UTH: University of Tokyo Hospital.

^c^ELECTRA: Encoder That Classifies Token Replacements Accurately.

^d^Not applicable.

**Table 7:** Performance of the model. Sentence-trained model evaluated using sentences.

Class	Tohoku-BERT^a^				UTH^b^-BERT				ELECTRA^c^		
	Precision	Recall	*F*_1_-score	Precision		Recall	*F*_1_-score	Precision		Recall	*F*_1_-score
Procedure adherence	0.798	0.798	0.796	0.802		0.750	0.768	0.754		0.844	0.796
Medicine	0.712	0.570	0.628	0.616		0.564	0.584	0.698		0.594	0.640
Resident	0.864	0.862	0.862	0.872		0.808	0.836	0.862		0.844	0.850
Nonmedical staff	0.744	0.686	0.714	0.692		0.670	0.678	0.774		0.674	0.716
Team	0.786	0.596	0.674	0.702		0.534	0.598	0.674		0.592	0.598
Environment	0.756	0.720	0.732	0.688		0.640	0.662	0.754		0.678	0.710
Organizational management	0.574	0.398	0.454	0.482		0.220	0.288	0.522		0.338	0.406
Macro *F*_1_-score	—^d^	—	0.694	—		—	0.631	—		—	0.674

^a^BERT: Bidirectional Encoder Representations From Transformers.

^b^UTH: University of Tokyo Hospital.

^c^ELECTRA: Encoder That Classifies Token Replacements Accurately.

^d^Not applicable.

**Table 8:** Exact match accuracy.

			Exact match accuracy		Exact match accuracy in test data with only multiple labels
**Tohoku-BERT^a^**					
	Report-trained model evaluated using reports	0.411		0.408	
	Sentence-trained model evaluated using reports	0.217		0.113	
	Sentence-trained model evaluated using sentences	0.656		0.318	
**UTH^b^-BERT**					
	Report-trained model evaluated using reports	0.389		0.378	
	Sentence-trained model evaluated using reports	0.202		0.113	
	Sentence-trained model evaluated using sentences	0.605		0.280	
**ELECTRA^c^**					
	Report-trained model evaluated using reports	0.399		0.394	
	Sentence-trained model evaluated using reports	0.198		0.095	
	Sentence-trained model evaluated using sentences	0.646		0.303	

^a^BERT: Bidirectional Encoder Representations From Transformers.

^b^UTH: University of Tokyo Hospital.

^c^ELECTRA: Encoder That Classifies Token Replacements Accurately.

## Discussion

### Principal Results

Our study constructed a multilabel classifier that used NLP models, including BERT and ELECTRA, to identify the factors contributing to medication-related incidents by nonmedical staff using incident reports of residential care facilities. Unlike previous studies that mainly focused on classifying incident types and harm severity in hospital settings [24-27], our approach focused on the complex factors involved in medication-related incidents involving nonmedical staff. This complexity often renders accurate classification challenging. To our knowledge, our study is the first to demonstrate the potential contribution of NLP technology in extracting incident factors and formulating measures from incident reports obtained from residential care facilities.

In our study, we identified and annotated 9-factor labels, leading to over 99% (2133) of the reports and more than 92% (6613) of the sentences being assigned these labels. Reports without factor labels merely described the conditions of the incident occurrences, lacking in-depth factor analysis. Hence, the 9-factor labels identified appear to be suitable for representing the contributory factors in medication-related incidents involving nonmedical staff in residential care facilities. In contrast, 2 specific labels, “resident family” and “medical staff,” were relatively limited. In residential care facilities in Japan, where nonmedical staff primarily provide medication assistance, the involvement of medical staff is limited, and family member participation is irregular. Consequently, these factors are less frequently represented, resulting in a small number of labels.

This study developed 2 types of models: 1 trained on individual sentences and the other on the entire reports. The report-trained model consistently outperformed the sentence-trained model, particularly achieving over 0.1 improvement in the *F*_1_-score for factors involving nonmedical staff. Thus, report-level training, which retains more contextual information than sentence-level training, significantly enhanced the model performance. The exact match accuracy was the highest using the sentence-trained model, exceeding 0.6. However, this accuracy dramatically decreased to approximately 0.3 when the test data were limited to those with multiple labels. This significant reduction underscores the prevalence of sentences with a single label, deeming it unsuitable for evaluation as a multilabel classifier. Therefore, we also evaluated the performance of the sentence-trained model using report units as test data; however, the performance was significantly inferior to that of the report-trained model. Conversely, training on the report data yielded an exact match accuracy of approximately 0.4, which remained stable across tests with multiple labels. These findings demonstrate the successful development of a multilabel classifier that can rather accurately classify multiple labels; nevertheless, the potential for further improvement exists. Further, 1 approach for model improvement involves analyzing label co-occurrences that are prone to errors and creating a data set of these label combinations from an existing data set for upsampling. Additionally, modifications to the model, such as incorporating other pretrained models or applying domain adaptation techniques, could also be effective methods for improving performance.

Within our report-trained multilabel classifier, the macro *F*_1_-scores of Tohoku-BERT and ELECTRA were notably similar, outperforming those of UTH-BERT, which had a lower score. This variation in performance was likely attributed to the characteristics of the pretraining data. Tohoku-BERT and ELECTRA were pretrained using the Japanese Wikipedia data, offering a broad range of general knowledge, whereas UTH-BERT was specifically pretrained on clinical texts. The lower classification performance for UTH-BERT may be attributed to the less specialized terminology included in the incident reports predominantly completed by nonmedical staff in residential care facilities compared to that in clinical texts.

Our analysis of the report-trained model’s performance across various labels revealed that, with the notable exceptions of organizational management and team factors, as assessed by UTH-BERT, the *F*_1_-scores consistently exceeded 0.6. Thus, the model accurately classified a broad spectrum of labels, thus demonstrating its effectiveness in automatically identifying incident factors from medication-related reports in residential care settings. However, classifying organizational management has proven to be more challenging. This difficulty can be attributed to the variability in the label assignment and the relatively limited number of labels in this category. Notably, the κ coefficient for the organizational management label was lower than that for the other labels, and the number of labels assigned to this category was also smaller. We assume that the low κ coefficient is partly attributed to the broader range of factors covered by this label, contributing to greater ambiguity compared to other labels. This highlights potential areas for the enhancement of the design and training process of our classifier.

Evaluation of the extrapolation of the constructed report-trained model confirmed that the *F*_1_-score was slightly inferior to that of its initial construction. This reduction was primarily due to the notably few reports used for extrapolation evaluation. Furthermore, the characteristics of the individual who completed the report could have influenced their performance, particularly since the report was from a hospital setting. Moreover, the extrapolation results showed that the model’s performance on reports involving care staff alone (*F*_1_-score=0.72) was higher than that on those involving nonmedical staff (*F*_1_-score=0.65). These findings indicate that the model is particularly effective in identifying factors in medication-related incidents involving care staff, suggesting specialization in extracting relevant information from such reports.

### Limitations

In total, 1 limitation of this study is the inclusion of data with a limited number of labels. Although 9 labels were assigned at the annotation stage, 2 specific labels, “resident family” and “medical staff,” were excluded from the multilabel classifier due to insufficient quantity. When a multilabel classifier that included these 2 labels was constructed, the *F*_1_-score for both labels was nearly zero. The *F*_1_-scores for the other labels remained almost unchanged compared to the case where the multilabel classifier was developed without including these 2 labels. Therefore, the impact of excluding these 2 labels is considered to be minimal. Furthermore, the performance of the model for each label tended to show higher *F*_1_-scores with a higher IAA and a greater number of labels. This problem can be resolved by increasing the number of incident reports and labels.

### Future Directions

Our model has the potential to streamline the identification of factors underlying medication-related incidents in residential care settings. This could result in a more effective planning of measures to prevent medication-related incidents. Moreover, it can offer nonmedical staff opportunities for learning and growth through prompt feedback following the occurrence of medication-related incidents.

### Conclusions

The multilabel classifier developed in this study can identify various factors associated with medication-related incidents based on incident reports from residential care facilities. This classifier can facilitate prompt analysis of incident factors, thereby contributing to risk management and the development of preventive strategies.

## Appendix

Multimedia Appendix 1 Hyperparameters of each model and label distribution of training and test data.

Multimedia Appendix 2 Extrapolation results of the fine-tuned Tohoku-BERT (Bidirectional Encoder Representations From Transformers).

## Abbreviations

IAA: interannotator agreement
BERT: Bidirectional Encoder Representations From Transformers
ELECTRA: Encoder That Classifies Token Replacements Accurately
NLP: natural language processing
UTH: University of Tokyo Hospital

## Data Availability

Permission to provide data to individuals other than this study’s investigators was not included under ethical approval and would require a new application for approval. The anonymized analyzed data may be obtained from the corresponding author upon reasonable request.
