# Supplementary material for: Construction of a Multi-Label Classifier for Extracting Multiple Incident Factors From Medication Incident Reports in Residential Care Facilities: Natural Language Processing Approach
Source: JMIR Med Inform. 2024 Jul 23;12:e58141. doi: 10.2196/58141 (PMC11303886; doi:10.2196/58141)
Supplement: Multimedia Appendix 1 [file medinform_v12i1e58141_app1.docx]

**Table S1.** The hyperparameter.

|  | Training data | Batch size | Epoch* | Learning rate |
| --- | --- | --- | --- | --- |
| Tohoku-BERT | Report | 4 | 32 | 2e-05 |
|  | Sentence | 4 | 32 | 2e-05 |
| UTH-BERT | Report | 4 | 32 | 2e-05 |
|  | Sentence | 4 | 32 | 1e-04 |
| ELECTRA | Report | 4 | 32 | 5e-05 |
|  | Sentence | 4 | 32 | 2e-05 |

* Early stopping was set for all the models.

BERT: Bidirectional Encoder Representations from Transformers

ELECTRA: Encoder that Classifies Token Replacements Accurately

**Table S2.** Label distribution of training data and test data.

| Label | Sentence | | Report | |
| --- | --- | --- | --- | --- |
|  | Training | Test | Training | Test |
| Procedure adherence | 933.6 | 233.4 | 792.8 | 198.2 |
| Medicine | 555.2 | 138.8 | 439.2 | 109.8 |
| Resident | 1964.0 | 491.0 | 1249.6 | 312.4 |
| Resident family | 36.8 | 9.2 | 26.4 | 6.6 |
| Non-medical staff | 1524.0 | 381.0 | 1063.2 | 265.8 |
| Medical staff | 18.4 | 4.6 | 16.0 | 4.0 |
| Team | 156.0 | 39.0 | 131.2 | 32.8 |
| Environment | 883.2 | 220.8 | 652.0 | 163.0 |
| Organizational management | 156.0 | 39.0 | 139.2 | 34.8 |

Numerical values indicate the average number of labels across the training and test data in a five-fold cross-validation setup.
