# Supplementary material for: Construction of a Multi-Label Classifier for Extracting Multiple Incident Factors From Medication Incident Reports in Residential Care Facilities: Natural Language Processing Approach
Source: JMIR Med Inform. 2024 Jul 23;12:e58141. doi: 10.2196/58141 (PMC11303886; doi:10.2196/58141)
Supplement: Multimedia Appendix 2 [file medinform_v12i1e58141_app2.docx]

**Table S1.** The extrapolation of the report-trained model, fine-tuned using Tohoku-BERT.

| Report-trained model | only care staff (31 reports) | | | | non-medical staff (136 reports) | | | |
| --- | --- | --- | --- | --- | --- | --- | --- | --- |
|  | Precision | Recall | F1-score | # of reports | Precision | Recall | F1-score | # of reports |
| Procedure  adherence | 0.62 | 0.62 | 0.62 | 8 | 0.70 | 0.42 | 0.53 | 33 |
| Medicine | - | - | - | 0 | 0.39 | 0.84 | 0.53 | 19 |
| Resident | 0.50 | 1.00 | 0.67 | 4 | 0.36 | 0.53 | 0.43 | 17 |
| Non-medical staff | 0.68 | 0.83 | 0.75 | 18 | 0.78 | 0.97 | 0.86 | 91 |
| Team | 1.00 | 1.00 | 1.00 | 2 | 0.10 | 0.50 | 0.16 | 6 |
| Environment | 0.67 | 0.67 | 0.67 | 6 | 0.68 | 0.67 | 0.68 | 39 |
| Organizational management | 1.00 | 1.00 | 1.00 | 4 | 0.70 | 0.44 | 0.54 | 16 |
| Micro F1-score |  |  | 0.72 |  |  |  | 0.65 |  |
